# Deep Gray Matter Demyelination Detected by Magnetization Transfer Ratio in the Cuprizone Model

**DOI:** 10.1371/journal.pone.0084162

**Published:** 2013-12-30

**Authors:** Sveinung Fjær, Lars Bø, Arvid Lundervold, Kjell-Morten Myhr, Tina Pavlin, Øivind Torkildsen, Stig Wergeland

**Affiliations:** 1 KG Jebsen Centre for MS-Research, Department of Clinical Medicine, University of Bergen, Bergen, Norway; 2 The Norwegian Multiple Sclerosis Competence Centre, Department of Neurology, Haukeland University Hospital, Bergen, Norway; 3 Neuroinformatics and Image Analysis Laboratory, Department of Biomedicine, University of Bergen, Bergen, Norway; 4 Molecular Imaging Center, Department of Biomedicine, University of Bergen, Bergen, Norway; 5 Department of Radiology, Haukeland University Hospital, Bergen, Norway; University of Illinois at Chicago, United States of America

## Abstract

In multiple sclerosis (MS), the correlation between lesion load on conventional magnetic resonance imaging (MRI) and clinical disability is weak. This clinico-radiological paradox might partly be due to the low sensitivity of conventional MRI to detect gray matter demyelination. Magnetization transfer ratio (MTR) has previously been shown to detect white matter demyelination in mice. In this study, we investigated whether MTR can detect gray matter demyelination in cuprizone exposed mice. A total of 54 female C57BL/6 mice were split into one control group () and eight cuprizone exposed groups (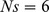). The mice were exposed to 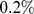 (w/w) cuprizone for up to six weeks. MTR images were obtained at a 7 Tesla Bruker MR-scanner before cuprizone exposure, weekly for six weeks during cuprizone exposure, and once two weeks after termination of cuprizone exposure. Immunohistochemistry staining for myelin (anti-Proteolopid Protein) and oligodendrocytes (anti-Neurite Outgrowth Inhibitor Protein A) was obtained after each weekly scanning. Rates of MTR change and correlations between MTR values and histological findings were calculated in five brain regions. In the corpus callosum and the deep gray matter a significant rate of MTR value decrease was found, 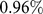 per week (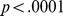) and 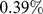 per week (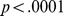) respectively. The MTR values correlated to myelin loss as evaluated by immunohistochemistry (Corpus callosum: 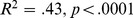. Deep gray matter: 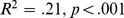), but did not correlate to oligodendrocyte density. Significant results were not found in the cerebellum, the olfactory bulb or the cerebral cortex. This study shows that MTR can be used to detect demyelination in the deep gray matter, which is of particular interest for imaging of patients with MS, as deep gray matter demyelination is common in MS, and is not easily detected on conventional clinical MRI.

## Introduction

Multiple sclerosis (MS) is a chronic inflammatory demyelinating disease of the central nervous system (CNS), characterized by demyelinated lesions, with a varying extent of remyelination. Conventional magnetic resonance imaging (MRI) shows hyperintense T2-weighted lesions and T1-weighted gadolinium-enhanced lesions in case of ongoing inflammatory disease activity. MRI is sensitive for detecting new white matter MS lesions. MS has traditionally been considered a white matter disease, but histopathology studies have shown that MS-pathology is frequent in the gray matter as well [1]. The extent of MS-gray matter demyelination has been determined in immunohistochemical studies, which found a similar proportion of demyelinated area in gray matter as in white matter. The true extent of gray matter demyelination has not been detected by MRI, as conventional MRI techniques have a very low sensitivity to gray matter demyelination, in particular to purely cortical lesions. This may be one of the contributors to the so-called clinico-radiological paradox, the weak correlation between total lesion load found in conventional MRI and clinical disability [2].

Many approaches have been investigated in order to overcome the clinico-radiological paradox, including spatial-pattern analysis of lesions in conventional MR images [3], diffusion tensor imaging in normal appearing white matter [4], [5], spinal cord MRI [6], T1-mapping [7], high-field MRI [8], double inversion recovery for detecting gray matter lesions [9], ultrashort echo time MRI for directly detecting myelin [10] and, as is the focus of this paper, indirectly detecting myelin content with magnetization transfer imaging.

Magnetization transfer ratio (MTR) has been introduced as a semi-quantitative measure of myelin, where the relative difference between the signal intensity of two MR images is calculated. The first of the two images is a conventional MR image, while the second image is acquired after application of an off-resonance pulse [11]. MTR, being sensitive to myelin loss, has been shown to decrease both in and near white matter MS lesions, which is suggestive of myelin loss [12]–[14]. MS patients have been shown to have lower average brain MTR than controls, and the different MS-phenotypes have different MTR distributions in the brain [15]. In MS, changes in gray matter are detected by MTR, and may predict disease progression in the long term [16], [17]. Re- and demyelination are not the only processes affecting MTR, as both inflammation and edema will typically decrease MTR values [18].

The cuprizone model is an experimental model of toxic de- and remyelination in mice. Earlier studies using MTR on the cuprizone model have shown significant changes in the corpus callosum during demyelination and remyelination [19]–[21]. The purpose of the present study was to examine whether the conventional MTR technique, as implemented on the standard preclinical systems, is sensitive enough to detect changes in myelin content in gray matter regions of the mouse brain during cuprizone exposure. For that purpose, we followed MTR changes in different regions of the brain over time, and then correlated these changes to the findings from histopathology.

## Materials and Methods

### Mice

A total of 54 female C57BL/6 mice (Tacomic, Tornbjerg, Denmark) were acquired at 7 weeks of age with a mean weight of 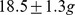 During the acclimatization and experimental period, they were housed, 6 per cage, in Macrolon IVC-II cages (Scanbur, Karlslunde, Denmark) in standard laboratory conditions: light/dark cycles of 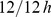, cage temperature of 22.3±1°C, relative humidity of 53.1±5% and 75 air changes per hour. They had ad libitum access to normal mouse chow (Rat and mouse No. I maintenance diet from Scanbur, Special Diet Services, Karlslunde, Denmark) and tap water during the acclimatization period of one week. Cage maintenance was performed once weekly by the same individuals throughout the study period. The experiment was conducted in accordance with the Federation of European Laboratory Animal Science Associations (FELASA) recommendations, and the protocol was approved by the Norwegian Animal Research Authority.

### Cuprizone Administration

After acclimatization, 48 mice were exposed to cuprizone for up to six weeks by adding 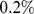 (w/w) cuprizone (bis-cyclohexanone-oxaldihydrazone, Sigma Aldrich, St. Louis, MO, USA) to the ordinary mouse chow, followed by two weeks of ordinary mouse chow. The control group consisted of six mice assigned to ordinary mouse chow.

### MRI Protocol

The MRI experiments were performed on a 7 Tesla horizontal bore magnet (Pharmascan 70/16, Bruker BioSpin, Bruker Corporation, Germany) using a 23 *mm* ID mouse-head linear volume resonator. All animals were scanned before cuprizone exposure (*N* = 54). The control group (*N* = 6) and a cuprizone-exposed group (*N* = 6) were scanned weekly during and two weeks after cuprizone exposure. Mice in the 7 other cuprizone groups (*N* = 6 in each group) were scanned at baseline, and immediately prior to being sacrificed for histopathology. Table 1 gives a schematic overview of the study setup. To assess remyelination, one cuprizone group (*N* = 6) was allowed to remyelinate for two weeks after removing cuprizone from the chow. These mice were scanned three times, at baseline, after six weeks of cuprizone exposure, and two weeks after terminating cuprizone exposure. Baseline scanning was done over the span of five days prior to cuprizone exposure. Each follow-up scanning was done over the span of two days. The control group and the cuprizone group that was scanned weekly were always scanned on the same day. During scanning, the mice were anesthetized by 1.5% isoflurane in O2, and the body temperature and respiratory frequency were monitored and kept constant at 37±1.5°C, and 80±20 respiratory cycles/min, respectively. The geometry was identical for all scans: 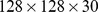 matrix size, 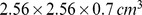 FOV, giving 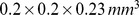 resolution. For the MTR acquisition, a FLASH sequence was used, with (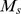) and without (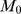) an offset magnetization transfer saturation pulse (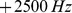 off resonance, Gaussian shaped, 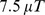 strength, 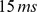 duration), 8 averages, 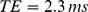, 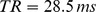 and *flip angle* = 10°. A T2-weighted (T2w) RARE image was acquired with 1 average, 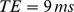, 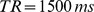 and a RARE factor of 16. Total scan time per animal was 26 minutes.

**Table 1:** Study setup.

Group	W0	W1	W2	W3	W4	W5	W6	W7	W8
Controls	S	S	S	S	S	S	S		HS
0	S	CS	CS	CS	CS	CS	CS		HS
1	S	HCS							
2	S	C	HCS						
3	S	C	C	HCS					
4	S	C	C	C	HCS				
5	S	C	C	C	C	HCS			
6	S	C	C	C	C	C	HCS		
7	S	C	C	C	C	C	CS		HS

Schematic representation of the study setup. Each cell in the table represent what happened to a group of animals at the given week, from baseline (W0) to week 8 (W8). S denotes that MR scans was obtained. C denotes cuprizone exposure. H denotes that the animals was sacrificed for histology. Groups 0–7 are the cuprizone exposed animals.

### MRI Analysis

Images were analyzed using in-house software written in Matlab (R2012a; The Mathworks, Natick, MA). The Magnetization transfer ratio (MTR) was calculated using the following formula voxel-wise: 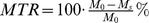. A semi-automatic method was used to segment the brains into anatomical regions. An experienced physician manually drew an anatomical segmentation map consisting of 5 brain regions (corpus callosum, deep gray matter, olfactory bulb, cerebellum and cerebral cortex) of one subject at baseline using the T2w image. This map was superimposed on all other subjects by linearly co-registering the T2w images using the Statistical Parametric Mapping 8 package (SPM8; Wellcome Trust Center for Neuroimaging, London, England; http://www.fil.ion.ucl.ac.uk/spm). Some cuprizone exposed animals experienced hydrocephalus, expanding the ventricles. It was assumed that this primarily affected the brain locally, and that the problem could be mediated by excluding ventricle voxels from the analysis. The cerebral spinal fluid is much more intense than brain matter. By a simple threshold, the same for each individual, ventricle masks were generated for each individual scanned brain. The mean MTR value was calculated in each region. To compensate for non-biological weekly variation in MTR, the cross-subject mean was calculated region-wise for the control group and subtracted from all subjects at that week. Prior to statistical analysis, a z-score outlier correction with three standard deviations was applied to the MTR data.

### Preparation of Brain Tissue

Mice were asphyxiated with 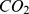, the brains were removed and fixated in 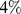 paraformaldehyde for at least 7 days, then paraffin embedded. Seven 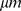 serial sections between areas 165 and 195 in the mouse brain atlas (http://www.hms.harvard.edu/research/brain) were analyzed. For immunohistochemistry, the sections were dewaxed and rehydrated before antigen retrieval in citrate buffer (pH 6.2). Sections were immunostained for myelin with anti-Proteolipid Protein antibody (PLP, Serotec, Oxford, UK) and for mature oligodendrocytes with anti-Neurite Outgrowth Inhibitor Protein A (NOGO-A, Chemicon, Temecula CA, USA). Sections were blocked with peroxidase blocking solution (Dako, Glostrup, Denmark), and visualized with EnVision 3.3-diaminobenzidine (Dako, Glostrup, Denmark). The tissue sections were counterstained with hematoxylin. For each antibody, omission of the primary antibody served as negative control. Normal brain tissue from the healthy controls served as positive controls.

### Characterization of Brain Tissue

**Figure 1:**
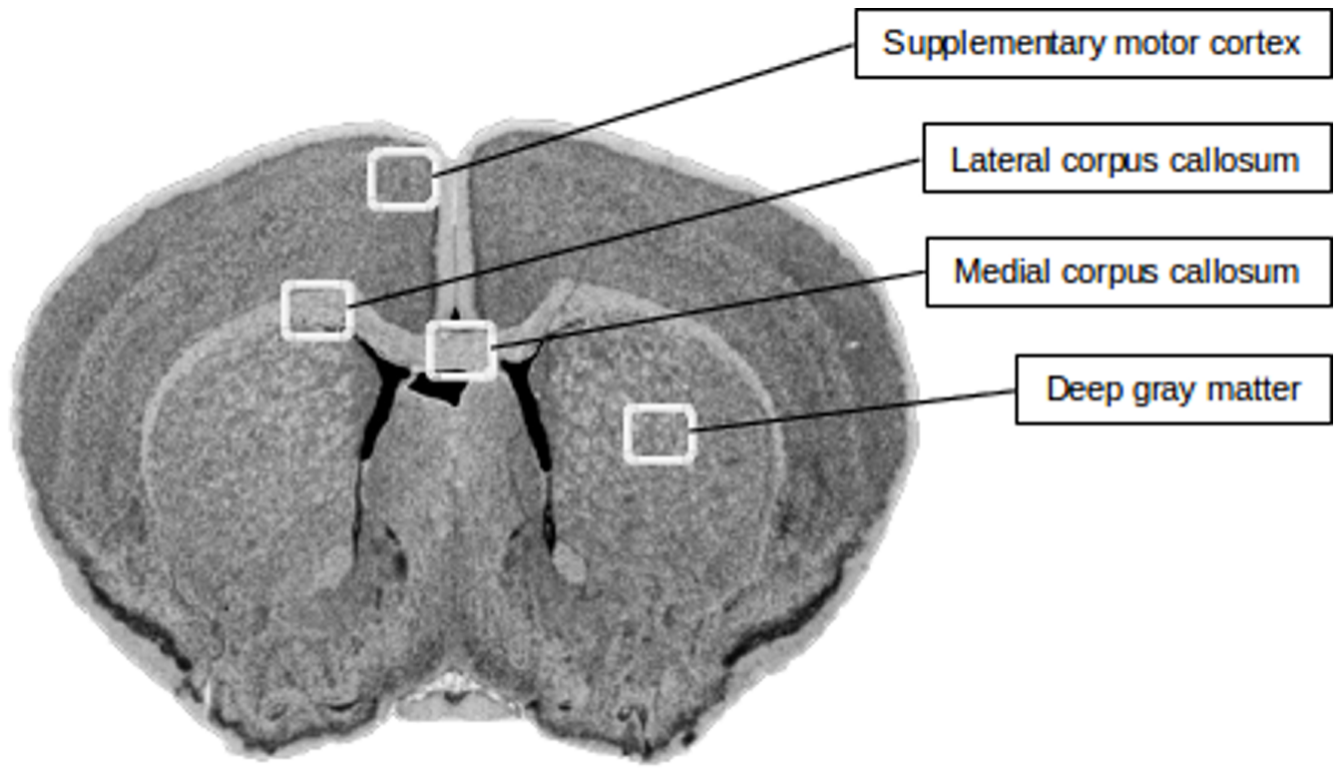
Tissue sections. Regions of the mouse brain where tissueimmunohistochemically analyzed for content of myelin (PLP) and oligodendrocytes (NOGO-A) were analyzed.

In tissue sections immunostained for PLP, medial and lateral regions of the corpus callosum, cerebral cortex and deep gray matter (Figure 1) were photographed with identical exposure settings at 40x magnification (Leica DMLe with Leica DC300 camera). Grayscale images were thresholded in order to avoid quantitative registration of low-intensity background staining. The area of PLP immunopositivity in each image was determined using Image processing and analysis in ImageJ (U. S. National Institutes of Health; Bethesda 2009), and expressed as the percentage of pixels, or relative area, in each image with an intensity within the threshold values. The number of oligodendrocytes (NOGO-A immunopositive cells) was counted in an area of 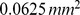 medial and lateral regions of the corpus callosum, in the cerebral cortex and the deep gray matter, using an ocular morphometric grid, and expressed as density (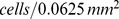).

### Statistical Analysis

Statistical analysis was done using R (RCore2012). The study design is longitudinal. To take into account dependent changes over time and repetitive measurements, a Linear Mixed Effects Regression (LMER) model was used [22]. From previous studies we expect the demyelination to be close to linear during the six weeks of cuprizone exposure [23], [24]. From this, a LMER model was constructed allowing for linear MTR differences between cuprizone exposed group and control group. In addition, a constant term for estimating remyelination after cuprizone exposure end was included. The random effects was modelled lineary. Giving the following LMER model:
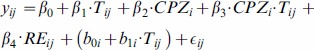




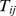
: Time in weeks. 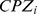: Group label variable. 1 for cuprizone exposed animals, 0 for control animals. 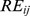: Remyelination tagging variable. 1 for cuprizone exposed animals in week 8. 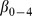: Fixed effects coefficients. 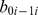: Random effects coefficients. : Random error/residual term. 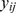: Response value (MTR value). Subscript 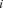 refers to animal. Subscript 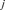 refers to examination nr.

The model was fit separately for each of the five brain regions using maximum likelihood. The model allows for a linear comparison between the control- and cuprizone animals during cuprizone exposure and during remyelination, two weeks after terminating cuprizone exposure. The fixed effects 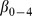 is the fitted variables. 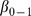 linearly fits the MTR value difference in the control animals, where both are zero by design. 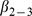 linearly fits the MTR value difference for the cuprizone animals, where 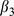 is of most interest as it gives the rate of MTR change during demyelination. 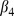 is a parameter for estimating MTR value difference change during the remyelination phase, where the rate of change during remyelination is given by 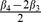. By linearly estimating random effects for each individual, which could include differences in individual response to the cuprizone as well as other less specific differences affecting the animals differently, this model is able to use the longitudinal information to separate individual deviations from group effects, making it more sensitive to group differences. A weakness in the model is that extreme values at one point in time can affect the estimated linear demyelination rate greatly. To account for this, it was required that the model gives similar result when run excluding data from a single time point. The degree of freedom from a LMER model is not clearly defined. To be able to present p-values, the degree of freedom was set to a lower bound of 11, giving conservative p-value estimates. The correlation between MTR measures and myelin content (PLP) as well as oligodendrocyte density (NOGO-A) was assessed using linear regression.

## Results

### Longitudinal MTR Change

**Figure 2:**
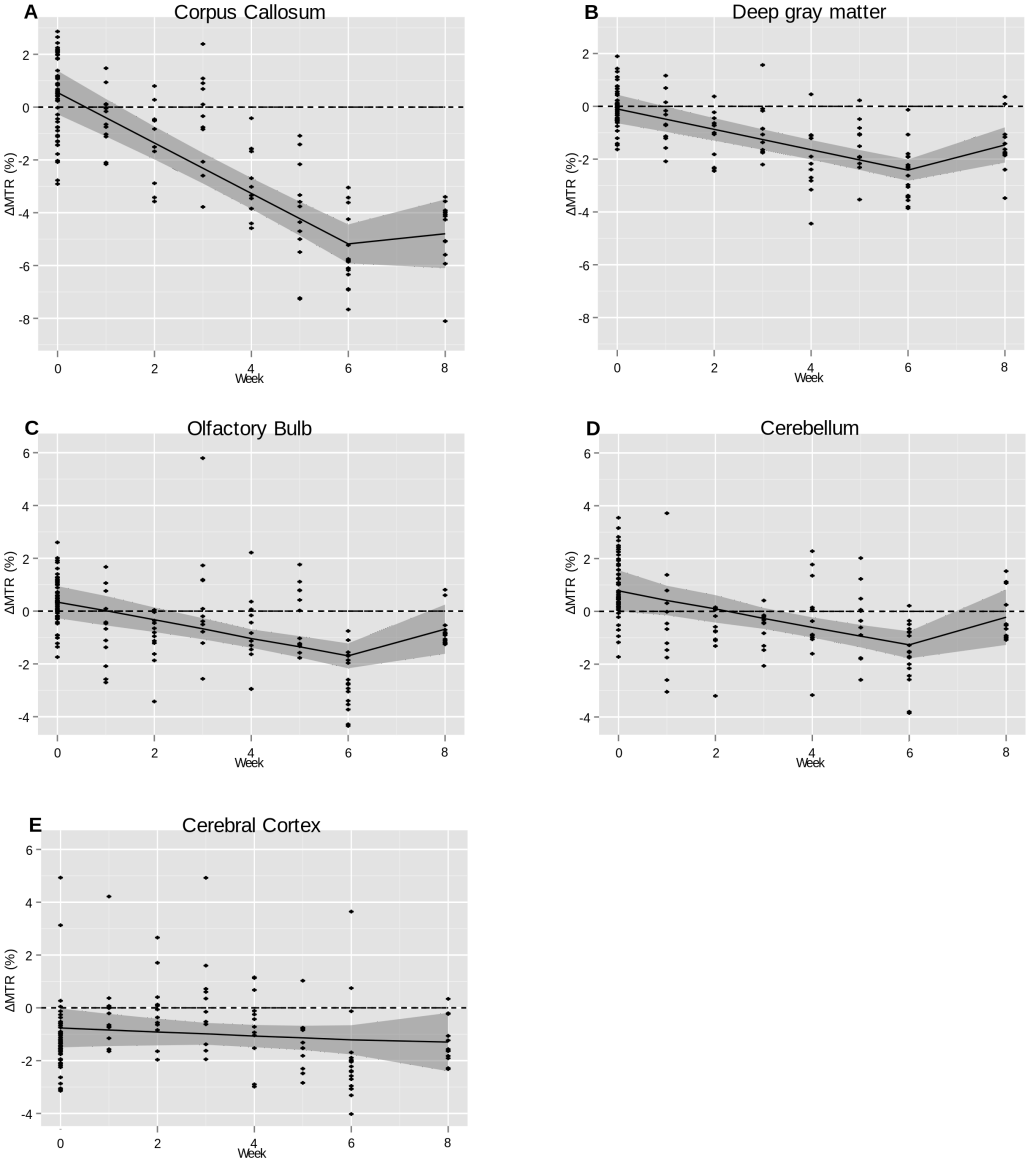
Longitudinal MTR values fitted with LMER. LMER regression of the difference in MTR values between cuprizone exposed animals and control animals over time. Dots represent single measures and the gray shaded area 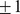 standard deviations of the LMER fit. A: The corpus callosum. B: The deep gray matter. C: The olfactory bulb. D: The cerebellum. E: The cerebral cortex.

In the corpus callosum, the MTR decreased significantly by 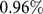 for each week of cuprizone exposure compared to the MTR values in controls (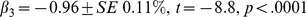) (Figure 2). In the deep gray matter, MTR in the cuprizone exposed group decreased significantly by 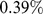 each week of cuprizone exposure compared to the MTR values in controls 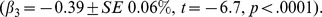 In the cerebellum 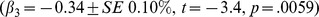 and in the olfactory bulb 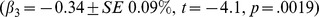, the LMER analyses also showed a significant decrease in the MTR during cuprizone exposure. However the baseline difference between control animals and the cuprizone exposed animals was larger than what would have been expected. When excluding the baseline MTR values from the LMER analysis, there were no significant differences in the MTR change between cuprizone exposed mice and controls in the cerebellum. In the olfactory bulb, the estimated weekly change in MTR was driven by the observed changes after six weeks of cuprizone exposure only. When excluding the MTR changes at week six, the MTR change was not significantly different between cuprizone exposed mice and controls. In the cerebral cortex (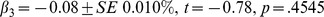), the MTR value did not differ between cuprizone exposed mice and controls. All estimates and their precisions are given in Table 2 and the regression curves are shown in Figure 2.

**Table 2:** LMER model parameters.

Area	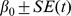	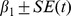	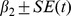	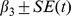	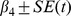
CC	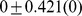	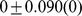	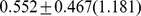	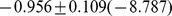	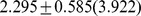
DG	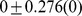	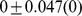	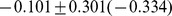	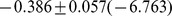	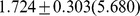
CE	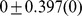	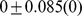	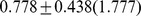	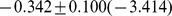	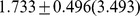
BO	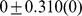	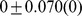	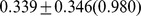	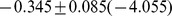	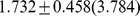
CO	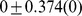	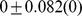	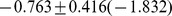	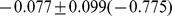	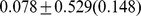

Estimated fixed effect coefficients from the linear mixed effects regression, with standard deviation and and t-value. Note that 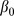 and 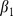 are 0 by design. CC: The corpus callosum. DG: The deep gray matter. CE: The cerebellum. BO: The Olfactory bulb. CO: The cerebral cortex.

MTR change after terminating cuprizone exposure was assessed in regions with significant change during cuprizone exposure, i.e. the corpus callosum and in the deep gray matter. After termination of cuprizone exposure, the rate of MTR increase was significantly higher in the corpus callosum (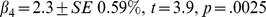) and in the deep gray matter (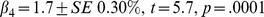) compared to during the cuprizone exposure. This corresponds to a remyelination rate of 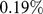 and 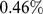, respectively.

### Longitudinal Histopathological Change

**Figure 3:**
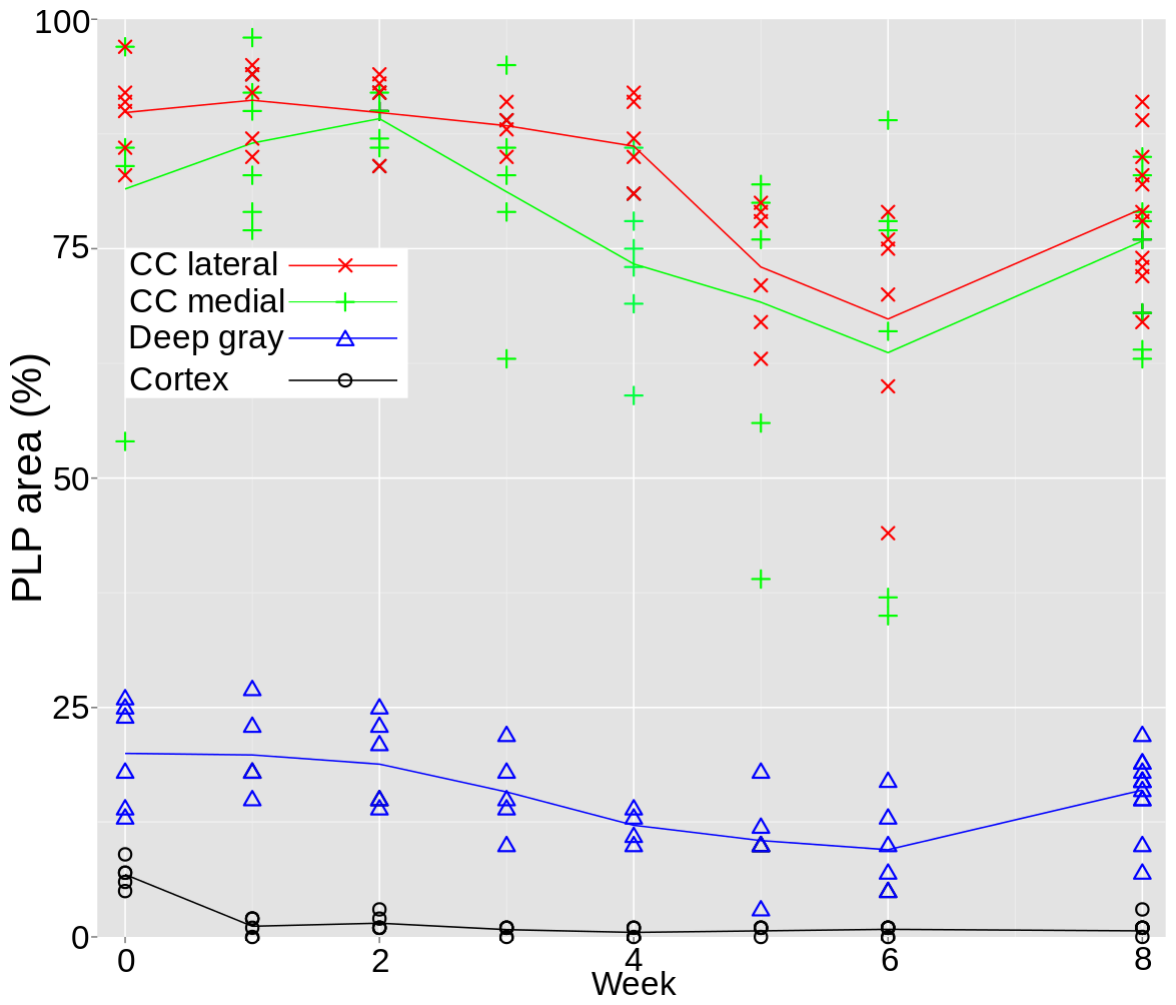
Longitudinal PLP area. Progression of myelin content over time, estimated from PLP stained tissue. Lateral in the corpus callosum (red 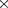), medial in the corpus callosum (green 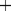), in the deep gray matter (blue 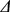) and in the cerebral cortex (black ).

**Figure 4:**
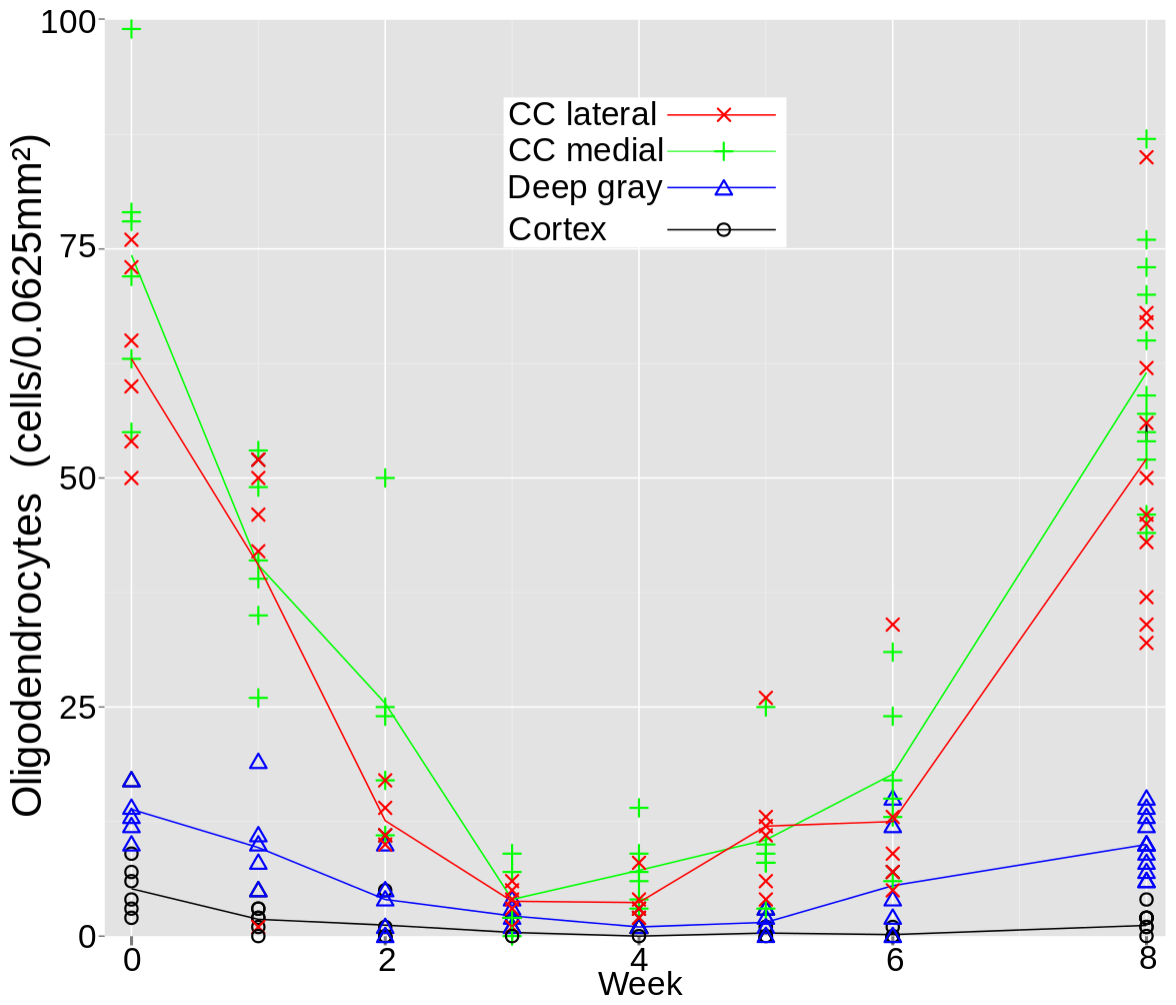
Longitudinal NOGO-A density. Progression of oligodendrocyte density over time, estimated from NOGO-A stained tissue. Lateral in the corpus callosum (red 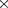), medial in the corpus callosum (green 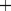), in the deep gray matter (blue 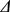) and in the cerebral cortex (black ).

As evaluated by PLP immunopositivity (Figure 3), there was a decrease in myelin content during cuprizone exposure in all examined regions. In the corpus callosum, the decrease became evident in week three. The lowest myelin content was detected during the last week of cuprizone exposure. In the deep gray matter, myelin loss was detected in week two and continued during the period of cuprizone exposure. In the cerebral cortex, an almost total loss of myelin was found after one week of cuprizone exposure. This loss was not regained. Oligodendrocyte density, assessed by NOGO-A immunopositive cells (Figure 4) showed a rapid decline in the corpus callosum from baseline starting at the beginning of cuprizone exposure, and continuing for three weeks. During the last three weeks of cuprizone exposure, the oligodendrocyte density increased, and was almost totally restored to baseline levels two weeks after terminating cuprizone exposure. In the deep gray matter, a decrease in oligodendrocyte density was found during the first four weeks of cuprizone exposure, with an increase during the last week of cuprizone exposure and two weeks after cuprizone exposure end. In the cerebral cortex there was a near total loss of NOGO-A immunopositive cells after three weeks of cuprizone exposure, and only a minor increase in oligodendrocyte density was observed two weeks after terminating cuprizone exposure.

### Histological Correlation to MRI Data

**Figure 5:**
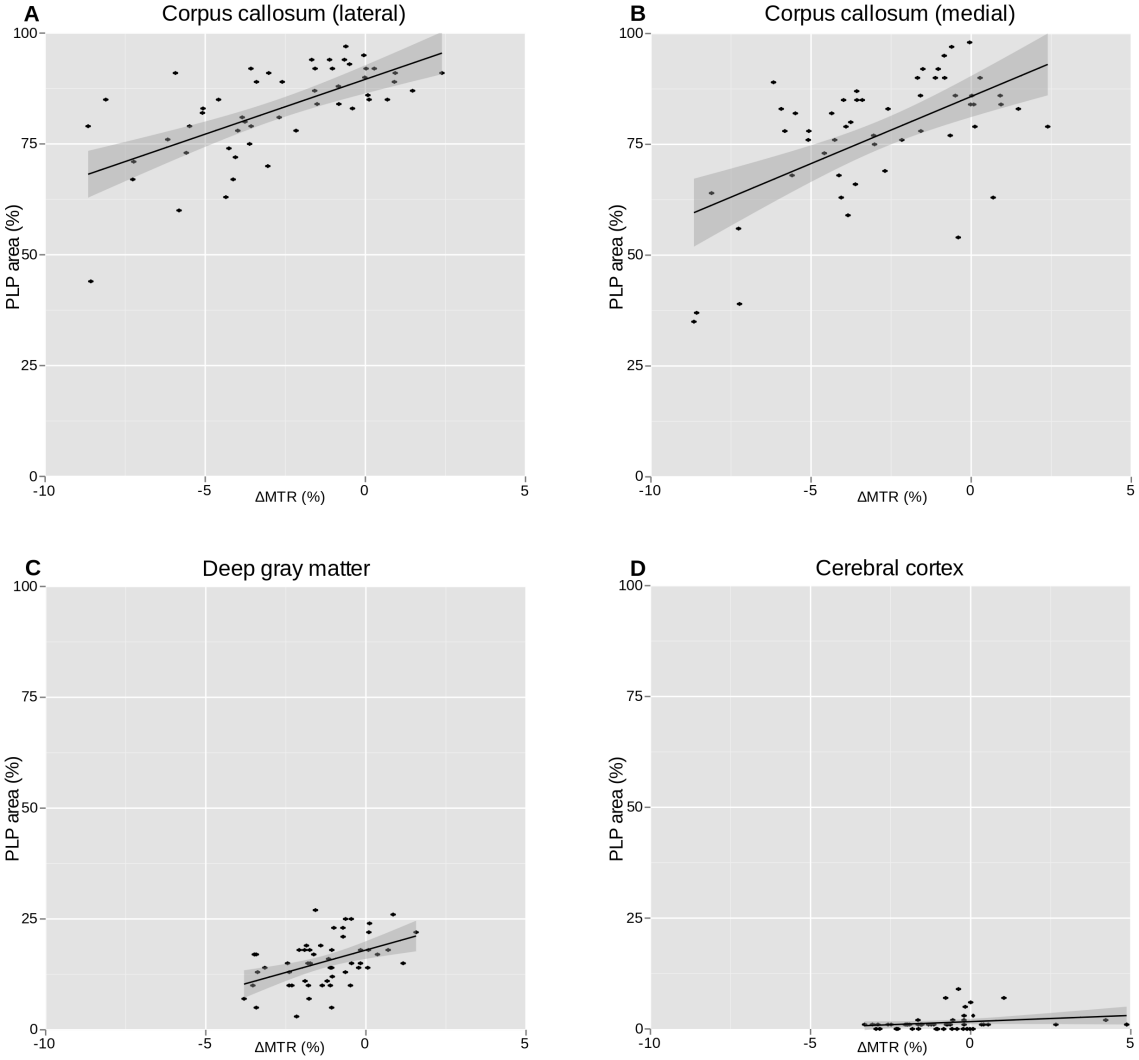
PLP area correlated to MTR value. Correlation between myelin content, estimated from PLP stained tissue, and MTR values. Dots represent animals, with MTR measurement taken imediately before being sacrificed for histological staining.

**Figure 6:**
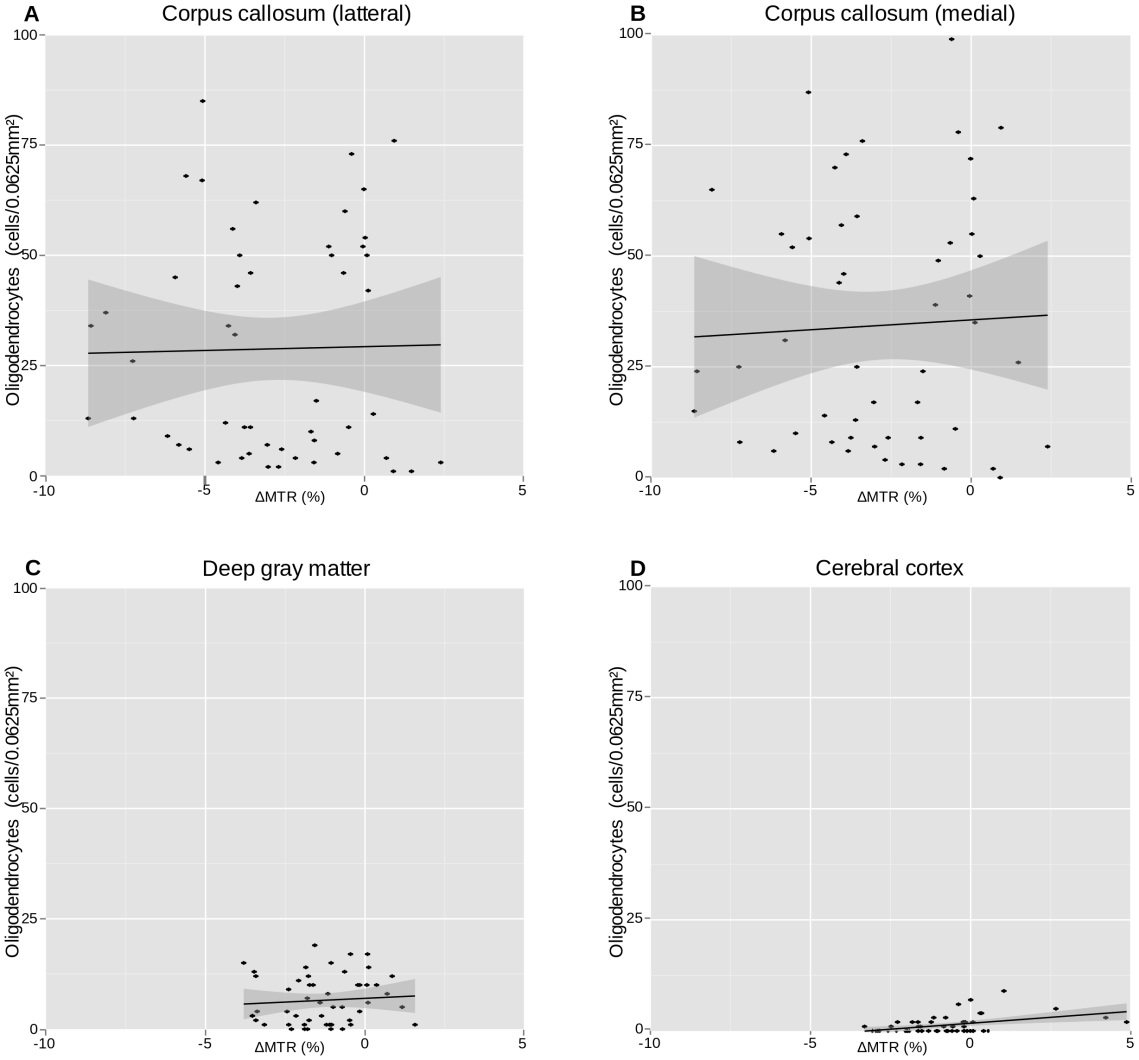
NOGO-A count correlated to MTR value. Correlation between oligodendrocyte density, estimated from NOGO-A stained tissue, and MTR values. Dots represent animals, with MTR measurement taken imediately before the animal was sacrificed for histological staining.

**Figure 7:**
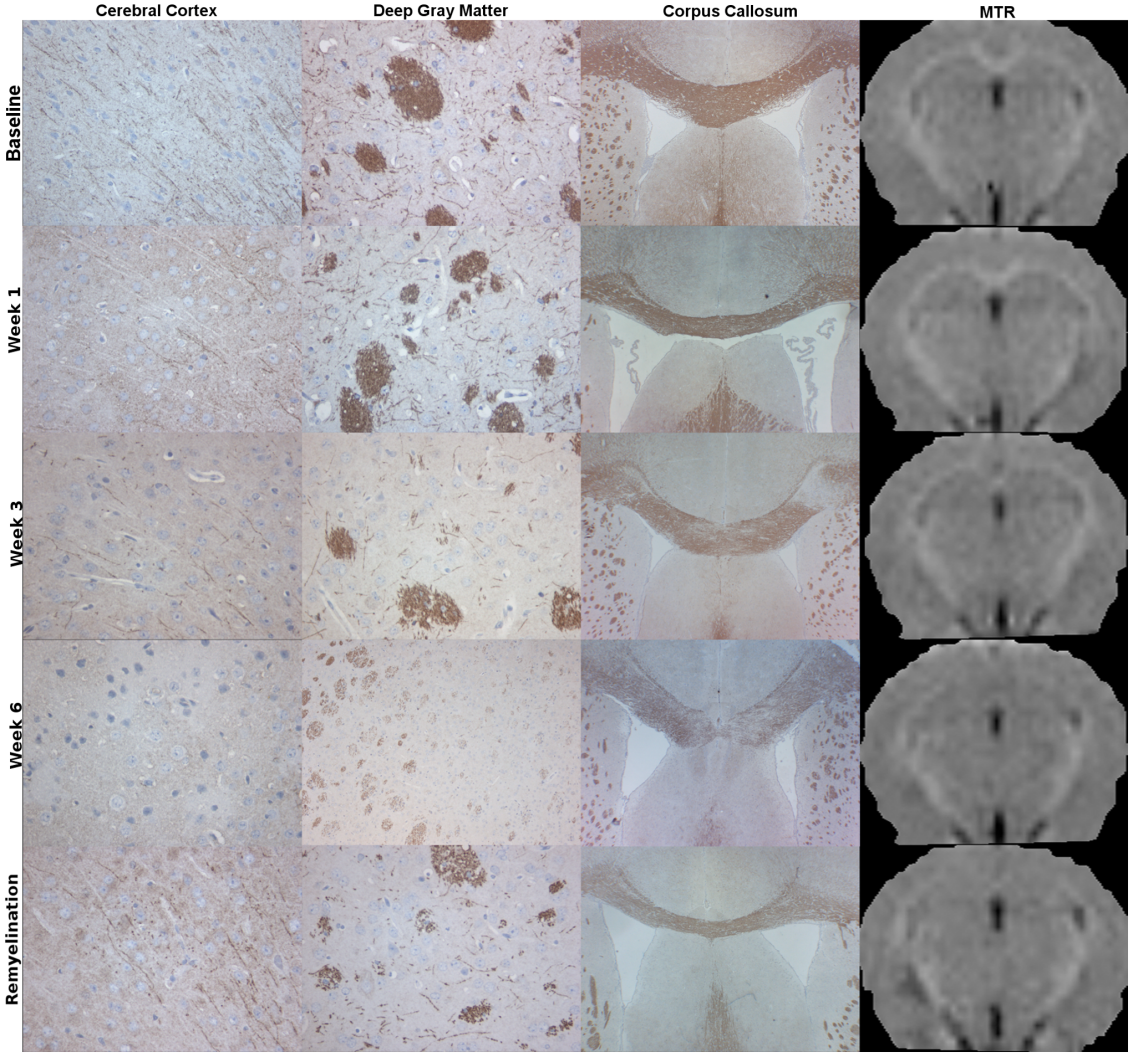
Myelin stained histology and MTR images. Representative images of PLP stained histology, from the cerebral cortex, the deep gray matter and the corpus callosum, and MTR image at baseline, 1,3 and 6 weeks of cuprizone exposure and two weeks after exposure end. Notice visible decrease of MTR values in the corpus callosum, corresponding to decrease seen in the PLP stained images.

In the corpus callosum, there was a significant correlation between the relative PLP immunopositive area in the medial and lateral corpus callosum and MTR values, with 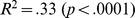 and 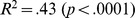, respectively, as shown in Table 3 and Figure 5. In the deep gray matter, a lower, but significant, correlation was found, with 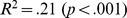. In the cerebral cortex there was no significant correlation between myelin content and MTR values. The oligodendrocyte density did not correlate with MTR values in neither the corpus callosum nor the deep gray matter (Figure 6 and Table 4). However, a significant correlation was found in the cerebral cortex, 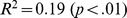. Figure 7 shows representative images of myelin (PLP) stained sections from the cerebral cortex, the deep gray matter and the corpus callosum, and MTR images from representative brains at baseline, after 1, 3 and 6 weeks of cuprizone exposure and two weeks after cuprizone exposure end.

**Table 3:** PLP - MTR correlation parameters.

Area	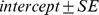	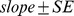	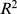	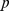
CC(m)	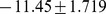	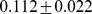	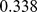	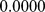
CC(l)	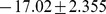	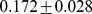	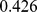	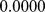
DG	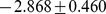	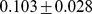	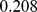	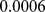
CO	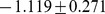	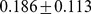	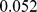	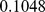

Parameters from regression analysis between MTR values and myelin content, calculated from PLP stained tissue. CC(m): Medial in the corpus callosum. CC(l): Lateral in the corpus callosum. DG: The deep gray matter. CO: The cerebral cortex.

**Table 4:** NOGO-A - MTR correlation parameters.

Area	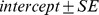	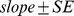	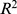	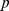
CC(m)	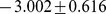	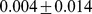	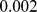	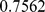
CC(l)	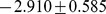	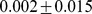	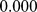	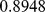
DG	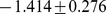	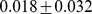	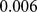	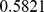
CO	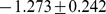	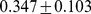	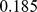	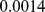

Parameters from regression analysis between MTR values and oligodendrocyte density, calculated from NOGO-A stained tissue. CC(m): Medial in the corpus callosum. CC(l): Lateral in the corpus callosum. DG: The deep gray matter. CO: The cerebral cortex.

## Discussion

The results of our study showed that there was a significant decrease in the MTR values in the cuprizone group compared to the controls in both the corpus callosum and the deep gray matter during demyelination. Significant MTR change in the cerebellum, the olfactory bulb, and the cerebral cortex was not detected. The MTR findings correlated significantly to myelin content as evaluated by PLP immunopositivity in both the corpus callosum and the deep gray matter, while there was no correlation between oligodendrocyte density and the MTR changes in these regions.

In the cuprizone exposed mice, there was extensive demyelination in the brain, with little or no T-cell mediated inflammation. This makes the cuprizone model ideal for investigating the sensitivity of the MTR technique to myelin content. There was a variation in the MTR values that could not have been explained by biology. The mean MTR value in the corpus callosum of the control group varied from 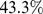 to 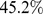 over the study period. The largest observed variation in one individual between two scan points was 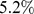, which is comparable with previous studies, where a variation of around 4% is seen in 9.4 T and 7 T preclinical systems [20], [21], [25] and over 10% in a 2.35 T preclinical system [19]. From a neurobiological perspective, only small variation, if any, was to be expected. We thus hypothesize that this variation was due to a systematic error, possibly originating from the MRI hardware. To circumvent this issue, we investigated the difference in the MTR values between the control and the cuprizone group, rather than the absolute MTR values. An inter-day variation in MTR values was also observed. This was most apparent during baseline scanning, during which 54 control animals were scanned over a period of five days. To partly control for this effect, the cuprizone group that was scanned every week was always scanned on the same day as the control group.

The slope of the MTR change calculated in the LMER model is sensitive to large group deviations at single time points. When determining whether a slope should be considered as a significant finding, it was required that the slope was significantly different from zero even when single time points were taken out of the calculations.

In the corpus callosum, there was a rapid decrease of the MTR values in the demyelinating phase. This is also visually confirmed in the MTR images, in which the corpus callosum became more hypointense each week. After six weeks of cuprizone exposure, the corpus callosum was no longer readily identifiable in the MTR images. After the termination of cuprizone exposure, the change in MTR significantly differed from that during the exposure, but it was not significantly increasing. On the other hand, in the deep gray matter, a significant decrease in MTR during cuprizone exposure was followed by a significant increase after the termination of cuprizone exposure. Unlike in the corpus callosum, this change was not visually obvious. In the cerebellum, the decrease in MTR values during cuprizone exposure was not considered to be significant, even though the LMER model gave a significant negative slope. The measured baseline MTR difference between the control and other, not yet cuprizone exposed groups was 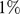, whereas a difference of near 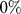 is to be expected. It is possible that this effect is seen due to day-to-day variation in the MTR values on the MRI scanner. When the analysis was performed solely on control animals and the cuprizone exposed animals scanned on the same day as the controls, or when omitting the baseline scan in the calculations, no significant MTR change was found in the cerebellum. In the olfactory bulb, the MTR decrease during cuprizone exposure was not found to be significant, even though the LMER model gave a significant negative slope. When we excluded the time point at week 6 of cuprizone exposure from the analysis, the slope was close to zero, i.e. 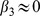. Finally, in the cerebral cortex, no significant change in MTR was seen during cuprizone exposure. The MTR values did not correlate to myelin content.

As evaluated in the PLP immunostained sections, the myelin loss in the cerebral cortex is almost complete, and occurs during the first week of cuprizone exposure. To obtain a significant change and PLP correlation in the LMER model, a continuous loss throughout the whole period of cuprizone exposure would be required. Thus, the failure to detect a significant MTR change in the cortex may be due to a limitation in the LMER model. A small, but significant, correlation between MTR and oligodendrocyte count was seen. As both oligodendrocyte count and myelin density were low in this region, small variation can lead to significant, but not real, correlation. The correlation was not present in the other regions. We observed a significant MTR change in the deep gray matter of cuprizone exposed mice. A difference in myelin density may contribute to the observed difference in MTR change between the cortical and deep gray matter, as the deep gray matter is more densely myelinated compared to cortical gray matter. Further, the proximity of these two regions to the ventricles may affect the MTR values differently. Failure to exclude the CSF from the analyzed regions would reduce the observed MTR change, as CSF typically has MTR values close to zero [26]. However, in order to minimize this, the threshold used for making the CSF mask was set low to include most voxels affected by partial voluming. Likewise, the MTR values are affected by oedema. In the brain of patients with MS, a typical difference between cortical gray matter and white matter lesions is the absence of macrophages and lymphocyte infiltrates in the cortex, with no oedema and an intact blood-brain barrier. Differences in the degree of oedema could theoretically contribute to the observed differences between the cortex and all other regions in our study. However, the cuprizone model induces demyelination with similar features as in human cortical MS lesions, with insignificant lymphocyte and microglia infiltration, and an intact blood-brain barrier, thus reducing the degree of oedema in the brain parenchyma significantly [27]. Therefore, cuprizone induced demyelination is a suitable model in the studies of MTR and its sensitivity to changes in myelin content.

In patients with MS, there are no differences the MTR values in normal appearing cortical and subcortical gray matter [28], [29]. Further, T2- and FLAIR MRI sequences also have low pathologic sensitivity for cortical and deep gray matter lesions, ranging from 3% to 38% [9], [30]. These regions are frequently affected in MS patients. The extent of subcortical gray matter involvement seem to correlate with cortical, but not subcortical demyelination, and it has been suggested that white matter axonal loss may be driven by pathology involving thalamocortical projections [31]. Cuprizone exposure in mice induces extensive demyelination both in white matter and in cortical and deep gray matter, and an interesting further investigation would be to study the correlation between demyelination and neuronal/axonal loss in these regions.

In a study of recently deceased patients with MS, Chen et al. showed that demyelinated cortex had lower MTR values than myelinated cortex [32]. In another clinical study of MS patients over 13 years, Filippi et al. showed that decreased gray matter MTR values predicted cognitive deteriation in MS patients [33].

In previous animal studies, it has been shown that the MTR technique can be used to detect pathological changes in the corpus callosum [19]–[21], [25] and within white matter lesions [34], [35]. Aharoni et al. use an atlas-based segmentation to show significant MTR differences between two different EAE models and control animals in many different regions of the mouse brain. To our knowledge, similar work has not been done in the cuprizone model, where the corpus callosum has been the main focus of study. Using a 2.35 T preclinical magnet, Merkler et al. [19] showed a decrease in the MTR values in the corpus callosum after 6 weeks of cuprizone exposure, followed by an increase 6 weeks after terminating cuprizone exposure. Using a 9.4 T magnet, Zaaraoui showed a strong correlation between the MTR values and myelin content in the corpus callosum, using thalamus as a reference [20].

Other techniques have also been proposed to quantify gray matter myelin content. Using an Ultrashort Echo Time (UTE) technique, Wilhelm et al. showed that myelin can be directly quantified using T2* mapping [10]. Although promising, the technique is in early stages of development and validation, and not readily available on clinical scanners. Using a 7 T preclinical scanner, Thiessen et al. showed that both T1-map and the bound proton fraction, f, from quantitative magnetization transfer, can detect differences between control animals and cuprizone exposed animals in the cortex ex vivo [36]. Currently, these techniques might not be clinically feasible due to high acquisition times. Using a 9.4 T preclinical scanner, Aharoni et al. showed that DTI can detect gray matter changes in two different EAE-models using the apparent diffusion coefficient (ADC) [25]. This technique is readily available at clinical scanners, but is not myelin specific, as ADC is as much influenced by axonal loss and inflamation as by demyelination.

In this study we have shown that the MTR technique can detect changes in myelin content in areas with moderate to high myelin content, while in areas with low myelin content, as in the cerebral cortex, the MTR technique used in this study was not sensitive enough to detect any changes. The MTR technique can be used to detect demyelination in the deep gray matter, which is of special interest for imaging of patients with MS, as deep gray matter demyelination is common in MS, and is not easily detected on conventional clinical MRI.
