# Correction: Deep Gray Matter Demyelination Detected by Magnetization Transfer Ratio in the Cuprizone Model

**DOI:** 10.1371/journal.pone.0111828

**Published:** 2014-10-22

**Authors:** The PLOS ONE Staff

## Notice of Republication

This article was republished on September 30, 2014, to correct a typesetting error involving a missing equation in the Statistical Analysis subsection of the Materials and Methods section. The publisher apologizes for this error. Please download this article again to view the corrected version. The originally published, uncorrected article and the republished, corrected article are provided here for reference.

## Supporting Information

File S1 **Originally published, uncorrected article**
(PDF)

File S2 **Republished, corrected article**
(PDF)
